# Implication of the Mosquito Midgut Microbiota in the Defense against Malaria Parasites

**DOI:** 10.1371/journal.ppat.1000423

**Published:** 2009-05-08

**Authors:** Yuemei Dong, Fabio Manfredini, George Dimopoulos

**Affiliations:** W. Harry Feinstone Department of Molecular Microbiology and Immunology, Bloomberg School of Public Health, Johns Hopkins University, Baltimore, Maryland, United States of America; Stanford University, United States of America

## Abstract

Malaria-transmitting mosquitoes are continuously exposed to microbes, including their midgut microbiota. This naturally acquired microbial flora can modulate the mosquito's vectorial capacity by inhibiting the development of *Plasmodium* and other human pathogens through an unknown mechanism. We have undertaken a comprehensive functional genomic approach to elucidate the molecular interplay between the bacterial co-infection and the development of the human malaria parasite *Plasmodium falciparum* in its natural vector *Anopheles gambiae*. Global transcription profiling of septic and aseptic mosquitoes identified a significant subset of immune genes that were mostly up-regulated by the mosquito's microbial flora, including several anti-*Plasmodium* factors. Microbe-free aseptic mosquitoes displayed an increased susceptibility to *Plasmodium* infection while co-feeding mosquitoes with bacteria and *P. falciparum* gametocytes resulted in lower than normal infection levels. Infection analyses suggest the bacteria-mediated anti-*Plasmodium* effect is mediated by the mosquitoes' antimicrobial immune responses, plausibly through activation of basal immunity. We show that the microbiota can modulate the anti-*Plasmodium* effects of some immune genes. In sum, the microbiota plays an essential role in modulating the mosquito's capacity to sustain *Plasmodium* infection.

## Author Summary

The *Anopheles gambiae* mosquito that transmits the malaria-causing parasite *Plasmodium* has an intestinal bacterial flora, or microbiota, which comprises a variety of species. Elimination of this microbiota with antibiotic treatment will render the *Anopheles* mosquito more susceptible to *Plasmodium* infection. In this study we show that these bacteria can inhibit the infection of the mosquito with the human malaria parasite *Plasmodium falciparum* through a mechanism that involves the mosquito's immune system. Our study suggests that the microbial flora of mosquitoes is stimulating a basal immune activity, which comprises several factors with known anti-*Plasmodium* activity. The same immune factors that are needed to control the mosquito's microbiota are also defending against the malaria parasite *Plasmodium*. This complex interplay among the mosquito's microbiota, the innate immune system, and the *Plasmodium* parasite may have significant implications for the transmission of malaria in the field where the bacterial exposure of mosquitoes may differ greatly between ecological niches.

## Introduction

The malaria parasite has to go through series of complex developmental transitions within the mosquito vector before it can be transmitted to the human host. The major bottleneck for *Plasmodium's* development occurs during the ookinete invasion of the midgut epithelium, prior to the development of oocysts on the basal lamina [1]. The factors that are believed to contribute to parasite losses at this stage are digestive enzymes, the mosquito's immune defenses and the intestinal microbial flora [2]–[4].

Large communities of diverse microorganisms reside in insects with a major concentration in the intestinal sections [5]. While much research has been focused on the microbiota of the mammalian intestine and its role in defense against pathogenic microorganisms [6], studies of insect gut microbiota have mainly concentrated on the contribution of microbial endosymbionts to the host's nutritional homeostasis [5]. However, the microbiota of the insect gut has also been shown to play a pivotal role of preventing development of pathogens. Studies have reported the wide spread of various species of Gram-negative bacteria in the midguts of both laboratory-reared and field derived mosquitoes, and some of this flora has been associated with an inhibitory activity on the sporogonic development of the *Plasmodium* parasites in the mosquito midgut [7]–[11]. However, these studies have not identified the causal mechanisms through which the presence of bacteria negatively impacts on malaria parasite development.

Bacteria within the midgut lumen may directly interact with, and adversely affect, the different malaria parasite stages within the bloodmeal through the production of various enzymes and toxins or physical barriers that hinder the interaction between *Plasmodium* ookinetes and the midgut epithelium (reviewed in [12]). Alternatively, the effect of bacteria on parasite development may occur indirectly through alterations in the physiology of the mosquito host itself, possibly through induction of immune responses that are cross-reactive between bacteria and malaria parasites, and/or changes of host metabolism that would affect the composition of mosquito derived molecules that are essential for *Plasmodium* development. Some studies have indicated that some of the mosquitoes' immune factors induced by bacterial challenge are involved in the killing of parasites at the pre-oocysts development stages [13]–[15]. Indeed, a great overlap, at the functional level, between antibacterial and anti-*Plasmodium* immune responses has been observed and suggests that mosquitoes lack highly specific mechanisms for defense against malaria parasites, but are using their anti-bacterial mechanisms to limit *Plasmodium* infection [14],[16],[17]. A reasonable hypothesis is that the presence of bacteria activates the mosquito's antimicrobial immune responses and the synthesized antimicrobial peptides and other immune factors will act against co-infecting *Plasmodium* parasites.

Indeed, a complex interplay between the mammalian immune system and the intestinal microbiota is essential for protection from infectious pathogenic microorganisms [18]. Some intestinal microbial species induce innate immune effector molecules which can kill competing bacterial species, including pathogens (reviewed in [19]).

The composition of mosquito midgut microbiota is much less complicated than that of mammalian intestine microbiota which makes it as a good model for dissecting the dynamics between the host innate immune system, natural bacterial flora, and the pathogenic microorganisms. Besides, mosquitoes transmit a broad range of human parasitic and viral diseases, within which malaria is still one of today's most devastating infectious diseases. A better understanding of the roles of microbiota in the exploiting host immunity in defending against pathogens could potentially lead to the development of new malaria control strategies.

We have examined the influence of the mosquito's midgut microbial flora and the derived antibacterial immune responses on malaria parasite infection through a series of infection assays in conjunction with functional genomics analyses.

## Results/Discussion

### Composition of microbiota in *A. gambiae* mosquitoes

**Figure 1:**
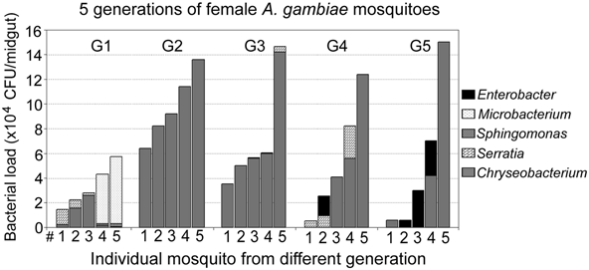
Distribution of bacterial loads and major species composition of midguts microbiota in 5 individual laboratory-reared 5-d-old female *A. gambiae* mosquitoes from 5 consecutive generations. The bacteria species were determined to be closely related to *Enterobacter asburiae*, *Microbacterium* sp., *Sphingomonas* sp., *Serratia* sp., and *Chryseobacterium meningosepticum*. G1 to G5 denotes generation 1^st^ to 5^th^.

To gain a better understanding of the potential fluctuations in microbial load and species composition between laboratory reared mosquitoes of different generations and within the same generation, we monitored the bacterial loads and species composition in individual five-day-old female *A. gambiae* mosquitoes of five consecutive generations. In accordance to previous studies our results showed a great variability in both parameters [8], [9], [20]–[22]. Interestingly, these variations were also observed between mosquitoes originating from the same generation and cage (Figure 1). This intriguing pattern may in some way relate to the equally broad distribution of *Plasmodium* infection intensities among mosquitoes that have fed on the same gametocyte culture. On average, individual mosquitoes carried around 40,000 colony forming units (CFU). Similarly to previous studies, the majority of the isolated bacteria were Gram-negative suggesting that the midguts of mosquitoes have more optimal growth conditions for this type, especially those from the *Enterobacteriaceae* family. This strong bias is also likely to have been attributed, to some degree, to the LB agar– based aerobic culturing method that was used for these assays. Sequence analyses of the 16s ribosomal genes from morphologically distinct bacteria colonies identified the following five different species as dominant in all assayed generations: *Enterobacter asburiae* (98%), *Microbacterium* sp. (98%), *Sphingomonas* sp. E-(s)-e-D-4(2) (100%), *Serratia* sp. (99%) and *Chryseobacterium meningosepticum* (100%). The *C. meningosepticum* and *Serratia* sp. species were dominant within all five generations and the former was the most abundant, especially within the second generation. Other bacteria identified from different generations were: *Asaia bogorensis* (99%), *Bacillus subtilis* (99%), *Enterobacter aerogenes* (98%), *Escherichia coli* (91%), *Herbaspirillum* sp. (99%), *Pantoea agglomerans* (98%), *Pseudomonas fluorescens* (99%), *Pseudomonas straminea* (99%), *Phytobacter diazotrophicus* (97%) and *Serratia marcescens* (99%). Interestingly, when *C. meningosepticum* became the dominant bacterium of the midgut flora, the growth of other bacterial species, that could be cultured on LB agar, was usually limited suggesting that this species may possess some competitive advantages in the gut environment.

Our LB agar –based culture assays have some limitations in providing the complete picture of the composition of the mosquito midgut microbiota since a large fraction of bacteria are likely to be un-culturable, similarly to the human intestinal microbiota [23]. Future high throughput sequencing -based metagenomics approaches are likely to provide comprehensive information on the composition of the midgut microbiota. Nevertheless, as a proof of principle, our approach shows the great variations in both load and composition of the microbiota between different individuals and generations of insectary-reared mosquitoes.

### The mosquitoes' natural microbiota can influence their permissiveness to *Plasmodium* infection

**Figure 2:**
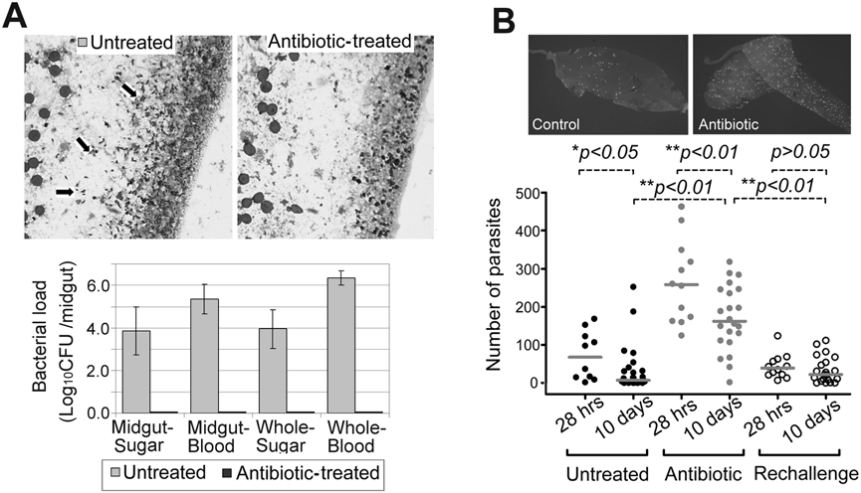
Antibiotic treatment eliminated the natural microbial flora from the mosquitoes' midguts. (A) The bacterial staining of the midguts of septic mosquitoes (Untreated) and aseptic mosquitoes (Antibiotic-treated), arrows indicating the bacteria (upper panel). Lower panel shows the bacterial loads from the homogenates of midguts (Midgut) or whole mosquitoes (Whole) from septic (untreated) or aseptic (antibiotic-treated) mosquitoes that had fed on either sugar or uninfected blood. (B) Aseptic mosquitoes (antibiotic-treated) became more susceptible to *P. falciparum* infection compared to the control septic mosquitoes. The upper panel shows IFA (immuno-fluorescence assay) slides of oocysts in the midgut epithelium which were stained with anti-Pfs25 8 days post infection. The lower panel shows the ookinetes numbers in the midgut epithelium (28 hrs) and oocysts counts (10 days) in uninfected septic, aseptic (Antibiotic), and antibiotic-treated mosquitoes that had been re-challenged with natural floral bacteria (Rechallenge). Points indicate the absolute value of parasites counts in individual mosquitoes, and horizontal black bars in each column represent the median value of parasites from three replicates. A Kruskal-Wallis test was used to determine the significance of oocysts numbers (*p<0.05* or *p<0.01*).

We assessed the impact of the mosquito's natural microbial flora on *P. falciparum's* capacity to establish infection through the removal of bacteria with antibiotic treatment, according to the established methodology [24],[25]. Provision of antibiotic through the sugar meal effectively eliminated all detectable bacteria from mosquitoes fed on either sugar or human blood (Figure 2A). The average bacterial load of sugar fed mosquito midguts was 10^4^ CFU, and those fed on blood contained as many as 10^6^ CFU (Figure 2A). After antibiotic treatment mosquitoes became aseptic and are referred as aseptic mosquitoes, while untreated mosquitoes are referred as septic. Aseptic mosquitoes were significantly more susceptible to *P. falciparum* infection, as a measure of oocysts numbers on the midgut, compared to the septic mosquitoes (*p<0.01*) (Figure 2B).

To gain a better understanding on the infection stage– specificity of this anti-*Plasmodium* action, we compared infection intensities between the septic and aseptic mosquitoes at two time points after ingestion of infected blood: at 28 hrs when ookinetes are still invading the midgut epithelium and at 10 days when all viable parasites have developed into oocysts on the basal side of the midgut epithelium. A significant larger number of ookinetes were found in the midgut epithelium of aseptic mosquitoes compared to the septic at 28 hrs after ingestion, suggesting that the bacteria-mediated anti-*Plasmodium* action has already taken place at pre-oocyst stages (*p<0.01*) (Figure 2B). Parasite losses during the transition from the ookinete to the oocyst stage were comparable between the septic and aseptic mosquito cohorts, suggesting that the presence of a microbial flora has little influence on parasite elimination at the early to late oocyst stages. A few aseptic mosquitoes displayed a very low infection level, while other had as many as 200 oocysts; this variation could be explained by potential differences in genetic background of individual mosquitoes. Future analyses will also address the impact of the microbiota on the later parasite stages in the mosquito.

To test whether the observed differences in infection levels between septic and aseptic mosquitoes could have been attributed to a direct interaction between the antibiotic and the parasite or mosquito, we re-challenged antibiotic treated aseptic mosquitoes with bacteria that had been previously isolated from midguts of adult females, prior to infection with *Plasmodium* (Figure 2B). The results from this assay suggested that the increased levels of oocyst infection in aseptic mosquitoes resulted from the absence, or at least a significantly decreased level, of bacteria, rather than a direct effect of the antibiotic itself on either the malaria parasites and/or the mosquito vector. The lower levels of oocysts in re-challenged mosquitoes compared to the untreated septic mosquitoes are likely to result from a higher bacterial load or the differences of the compositions of re-challenged bacteria to the natural flora.

Interestingly, the presence of the microbial flora influenced the mosquito's longevity upon *Plasmodium* infection; approximately 60% of the infected septic mosquitoes died by day 7 post-infection (fed with 1% *P. falciparum* gametocytes), in contrast to only 40% of the aseptic group despite an approximately 5-fold higher infection level (Figure S1 and Figure 2B). The mortality of the septic and the aseptic mosquitoes after feeding on non-infected blood did not differ significantly suggesting that the increased mortality of septic *Plasmodium* infected mosquitoes was caused in some way by the co-occurrence of bacteria and malaria parasites (data not shown). Interestingly, malaria-infected aseptic mosquitoes in which the midgut bacteria had been re-introduced exhibited reduced levels of mortality compared to untreated septic mosquitoes, possibly due to the presence of residual antibiotic in the tissues of these mosquitoes (Figure S1). This observation further supports the crucial impact of the microbiota on the mosquito's vector competence.

### Experimental exposure to bacteria influences the mosquitoes' permissiveness to *Plasmodium* infection

**Figure 3:**
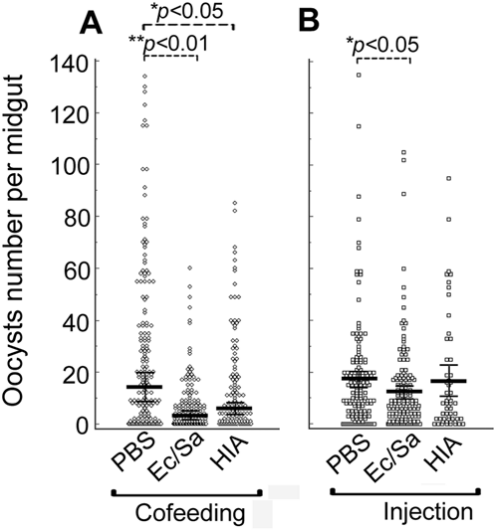
*P. falciparum* oocyst intensity in mosquitoes which had been co-fed with a mixture of live bacteria of *E. coli* and *S. aureus* (Ec/Sa) or heat-inactivated bacteria (HIA) in the blood meal, or mosquitoes that had been injected with live bacteria or heat-inactivated bacteria one day before the blood meal. Mosquitoes that had co-fed or been pre-injected with PBS served as controls. Points indicate the absolute value of oocysts counts in individual mosquitoes, and horizontal black bars in each column represent the median value of oocysts from three replicates where the narrow black bars above or below the median values indicate the standard errors. *p*-value was calculated through a Kruskal-Wallis test. (A) Oocysts counts from *P. falciparum* infected midguts which had been co-fed with bacteria. (B) Oocysts counts from *P. falciparum* infected midguts which had been pre-injected with bacteria one day before the infected blood meal.

In concordance with the previously described experiments, co-introduction of live or heat-inactivated bacteria with *P. falciparum* gametocytes in the midgut through feeding will result in a significantly decreased susceptibility to *Plasmodium* infection compared to the controls (Figure 3A); a 4-fold fewer oocysts developed in mosquitoes that had co-fed on live bacteria (*p*<0.01), and a 2.2-fold fewer oocysts developed in mosquitoes that had co-fed on heat-inactivated bacteria (HIA) (*p*<0.05) compared to the control mosquitoes. These and the previously described results suggest that the bacteria in the midgut lumen exert an anti-*Plasmodium* effect that could either involve a mosquito response or a direct interaction with the parasite. The frequency distribution of oocysts demonstrated that co-feeding with either live or heat-inactivated bacteria and pre-injection of live bacteria (discussed below) resulted in an over dispersion of oocysts, with the majority of mosquitoes having very few oocysts (Figure 3).

### Bacteria exert an indirect anti-*Plasmodium* activity

The decreased numbers of developing oocyst on the midguts of mosquitoes that had been exposed to bacteria suggested that the bacteria-mediated inhibitory activity on the parasite is acting prior to the oocyst stage. To test whether the negative effect of bacteria on malaria parasite development was to some degree attributed to a direct interaction by which the bacteria kill *Plasmodium*, we monitored *P. falciparum* development within the midgut lumen and epithelium of the four cohorts of mosquitoes (septic, aseptic, aseptic mosquitoes re-challenged with natural flora bacteria, or septic mosquitoes co-fed with experimental bacteria).

**Figure 4:**
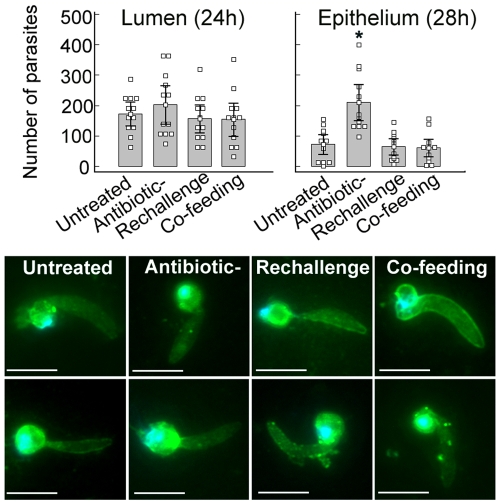
Ookinetes counts in the lumen or midgut epithelium of untreated septic, aseptic (Antibiotic), antibiotic treated mosquitoes that had been re-challenged with natural flora bacteria (Rechallenge), and mosquitoes that had been co-fed with live bacteria in the blood meal (Cofeeding) (upper panel). Points indicate the absolute value of ookinetes counts in individual midguts, and bars represent the mean value of ookinetes from two replicates where the standard errors are included. An asterisk denotes *p<0.01*, and *p*-value was calculated by a Kruskal-Wallis test. Lower panel: immuno-fluoresence staining of ookinetes in the midgut lumen 24 hrs post infection where midgut homogenates were stained with anti-Pfs25 antibody followed by Alexa Fluor 488-conjugated (green) goat anti-mouse antibody staining. Scale bars: 5 µm.

The prevalence of ookinetes in the blood-meal at 24 hrs after ingestion showed no significant difference between the four cohorts, suggesting that the bacteria had no effect on the pre-invasive stages. However, the number of ookinetes observed within the midgut epithelium was significantly higher in the aseptic mosquitoes, by approximately a 2.5-fold compared to the cohorts that contained bacteria (Figure 4, upper panel). The morphology of ookinetes was similar in the four cohorts (Figure 4, lower panel). These results suggest that the effect of bacterial exposure on mosquito susceptibility to *P. falciparum* occurs during ookinetes invasion, most likely through a mosquito response to the bacteria challenge which is likely to entail components of the mosquito innate immune system. Previous studies have indeed shown that the mosquito uses some of the same immune factors to combat bacteria and *Plasmodium* parasite infection [14],[26]. Another possibility is that the bacteria form a physical barrier which blocks the parasite's access to the epithelium; this is a common mechanism by which the vertebrate microbiota protect against pathogenic bacterial infection (reviewed in [19]). However, our current data does not directly support this hypothesis.

To provide further clues on this anti-parasitic mechanism we looked at the effect of hemocoel injected live or heat inactivated bacteria on the *P. falciparum* development. Injection of live bacteria at 24 hrs prior to feeding on a gametocyte culture resulted in a significant reduction of oocysts (*p*<0.05) while injection of heat inactivated bacteria had an insignificant effect on *Plasmodium* infection (*p*>0.05), compared to the PBS injected controls (Figure 3B). This result further supports that the anti-*Plasmodium* activity of bacteria is indirect and involves a response by the mosquito vector since the injected bacteria are unlikely to directly interact with the parasites that are confined within the midgut epithelium or under the basal lamina. It is more likely that the systemic infection will induce a battery of defense molecules in the hemolymph, from where they can attack the midgut-stage parasites on the basal side of the gut, or even within the epithelium by diffusion through the basal labyrinth. Indeed our previously published studies showed that injected bacteria induced a battery of anti-*Plasmodium* immune factors [14].

The stronger anti-*Plasmodium* effect of either injected or co-fed live bacteria, compared to heat inactivated bacteria, suggest that a factor which is more specific for live bacteria may be responsible for the inhibitory effect. Alternatively, the stronger effect of live bacteria may simply reflect their proliferative capacity which resulted in multiplication of their numbers to induce a much stronger immune response from the mosquito host.

### Mosquito genome-wide responses to microbial exposure

Mosquitoes, as all other higher organisms, are continuously exposed to a variety of microbes. And we have shown that this exposure, whether it originates from the midgut lumen or the hemolymph, can influence the mosquito's permissiveness to *P. falciparum* infection. We have also shown that this effect is likely to be mediated through a mosquito response to the bacterial exposure. To better understand this response we have performed a series of genome-wide expression analyses to assess the regulation of the mosquito transcriptome upon microbial exposure.

**Figure 5:**
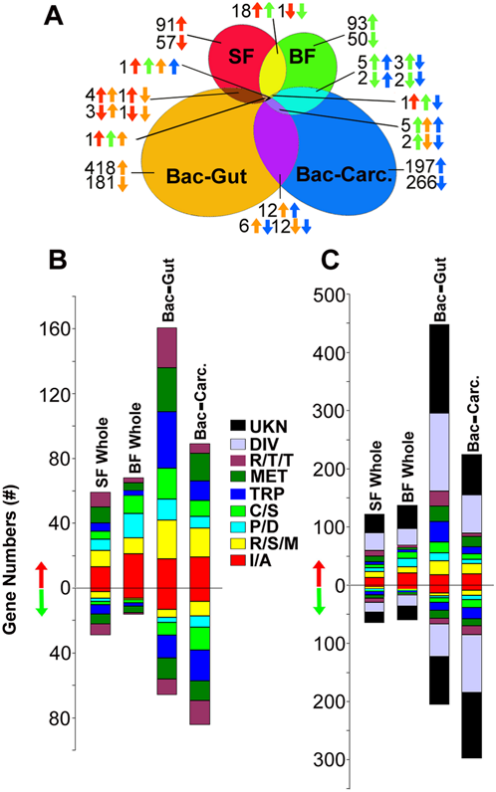
Global gene regulation at the different conditions of infection. (A) Comparison of transcript abundance between whole septic and aseptic mosquitoes after feeding on sugar (SF) or uninfected blood (BF), and in the midguts (Bac-Gut) or carcass tissues (Bac-Carc.) of mosquitoes 12 hrs post ingestion of uninfected blood supplemented with *E. coli* and *S. aureus* (substitution of bacteria with PBS as control). Colored arrows indicate genes that are up- or down- regulated in the corresponding treatment group. (B) Proportions and numbers of genes belonging to distinct functional groups which were up- or down- regulated in the corresponding treatment group. SF Whole: sugar-fed whole septic mosquitoes compared to aseptic ones; BF Whole: uninfected blood-fed whole septic mosquitoes compared to aseptic ones; Bac-Gut: mosquitoes midgut tissues 12 hrs post ingestion of experimental bacteria; Bac-Carc.: mosquitoes carcass tissues 12 hrs post ingestion of experimental bacteria (*E. coli* and *S. aureus*); I/A: putative immunity and apoptosis; R/S/M: oxidoreductive, stress-related and mitochondrial; C/S: cytoskeletal, structural; MET: metabolism; R/T/T: replication, transcription, translation; P/D: proteolysis, digestion; TRP: transport; DIV: diverse; UKN: unknown functions; gene functions were predicted based on Gene Ontology data and manual sequence homology searches. (C) Same as in (B), but also including genes of diverse functions (DIV) and unknown functions (UKN).

We used a microarray-based genome-wide gene expression strategy to compare transcript abundance between septic and aseptic adult female mosquitoes that had been fed on either sugar or non-infected blood (Figure 5 and Tables S2, S3). The presence of the endogenous bacteria flora in sugar fed mosquitoes resulted in the differential regulation of some 185 genes; 121 genes were up-regulated and 64 genes were down-regulated compared to antibiotic treated aseptic mosquitoes. A similar number of 195 genes were regulated by the presence of the endogenous microbial flora after feeding on non-infected blood; 137 genes were up-regulated and 58 genes were down-regulated (Figure 5A). The relatively small number of genes that were regulated as a consequence of the presence of the endogenous microbial flora most likely indicates a symbiotic relationship that has led to the adaptation of the mosquito to this flora. This hypothesis is strengthened by subsequent experiments that investigated the effect of ingested non-natural bacteria on the mosquito's transcriptome (see below). The mosquitoes' responses to natural microbiota when fed with either sugar or non-infected blood were quite divergent with only limited overlap in gene expression (Figure 5A), that comprised 21 induced and 1 repressed gene, corresponding to approximately 6.5% of the total regulated genes. However, one third of the commonly induced genes belonged to the immunity class.

The regulated genes represented a variety of functional classes with a general strong bias and over-representation of innate immunity genes (Figure 5B and 5C). Several of these immune genes have been previously shown to be transcriptionally-induced during malaria parasite infection, and to mediate anti-*Plasmodium* activity (Tables S2, S3). The septic mosquitoes displayed elevated expression of genes code for the antimicrobial peptides Cecropins 1 (Cec1) and 3 (Cec3), Defensin 1 (Def1) and Gambicin; the signal transducing serine proteases SP5, ClipA9, ClipA7 and ClipB8, and various pattern recognition receptors including AgMDL8, CTLMA4, FREP7 and FBN51, Tep4 and Tep5, Galectin 8, and PGRP-LB, PGRP-LC2 and PGRP-S3 [14], [27]–[31]. Surprisingly, the expression of the anti-*Plasmodium* factors FBNs 6, 9, and 36 were decreased in the septic mosquitoes (Tables S2, S3). The immune responsive Lysozyme c-1 (LYSC1) which previously has been linked to melanization reactions [32]–[34], was up-regulated in septic sugar-fed mosquitoes; lysozymes are key antibacterial factors. These results suggest that the natural microbiota play an important role in stimulating a basal immune activity which in turn is likely to contribute towards the determination of the mosquito's susceptibility to various pathogens, and hence their vectorial capacity. In fact a recent study has established that *Plasmodium* development is significantly more influenced by the mosquito's basal level immunity rather than the induction of immune responses upon parasite infection [35].

Of particular interest was the elevated expression of the peritrophic matrix protein gene *Ag-Aper1* in septic mosquitoes that had fed on either sugar or uninfected blood, and several other genes encoding proteins with peritrophin-like, laminin-EGF-like and chitin-binding like domains (Tables S2, S3) [36]. Ag-APer1 and proteins containing chitin-binding domains may function as structural components of the insect cuticle, the peritrophic matrix and/or as pattern recognition receptors. The elevated expression of *Ag-Aper1* in septic mosquitoes may indicate a role of the peritrophic matrix in protecting the epithelium from the infection of midgut flora bacteria. The natural microbial flora also stimulated expression of several metabolic genes involved in glycolysis, gluconeogenesis and sugar transport and this may relate to digestion of midgut bacteria that function as a food source for the mosquitoes [37] (Tables S2, S3). The genes exhibiting the greatest fold-differences in expression between septic and aseptic mosquitoes were of unknown function (Figure 5C).

To investigate the mosquito's global transcriptional response to exposure to non-natural midgut flora we compared transcript abundance between mosquitoes that had fed on blood supplemented with a mixture of both Gram-negative (*E. coli*) and Gram-positive (*Staphylococcus aureus*) bacteria and control mosquitoes that had fed on uninfected blood with PBS. These treatments resulted in a much broader response. The ingestion of these bacteria triggered the regulation of as many as 656 and 520 genes in the midgut and carcass, respectively (Figure 5 and Tables S4, S5). In the midgut, 458 genes were up-regulated and 198 genes were down-regulated. As expected, fewer genes were regulated in the carcass compared to midgut tissue which was in direct contact with the ingested bacteria; 224 genes were up-regulated and 296 were repressed.

Among the immune genes exhibiting differential expression between sterile-blood-fed and bacteria-blood-fed mosquitoes were several that have previously been shown to mediate anti-*Plasmodium* immune responses and to be transcriptionally up-regulated during *Plasmodium* parasite infection (Tables S4, S5). The ingestion of bacteria stimulated an elevated expression of genes code for the antimicrobial peptide IRSP1, the signal transducing serine proteases ClipB16, and inhibitor SRPN6 and SRPN7, and various pattern recognition receptors including AgMDLs 4, 6, and 7, CTL, CTLGA1, CTLGA3, and CTLMA6, FBNs 9, 20, 21, and 51, LRRD8, PGRP-LB, PGRP-LC2 and PGRP-S3, Tep11 and Toll6 [14], [38]–[42]. Only four immune genes, *SP5*, *TPX4*, *DCCE2*, and *FBN51* were induced by both the natural flora and the ingested non-natural bacteria, while *Tep*-like, *PGRP-LD*, and *FBN9* displayed an opposite pattern of regulation (Figure 5A and Tables S4, S5). As mentioned above, these differences are likely to reflect an adaptation of the mosquito to its natural microbial flora. Potential differences in the dosage of bacterial exposure may however also have influenced the quite different outcome.

### The natural microbiota stimulates basal immune activity that controls its proliferation

**Figure 6:**
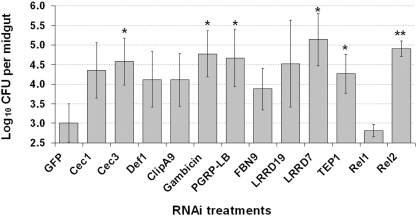
Immune gene-silencing influenced the bacterial loads of the mosquito midguts. Bars represent the mean values of total CFUs (log_10_ transformed) from 10 midguts, and standard error bars are included. *, *p<0.05*; **, *p<0.01*.

Depletion of several immune factors through RNAi-mediated gene silencing has been shown to result in a proliferation of bacteria in the hemolymph as a result of a compromised immune system [43],[44]. To test whether the immune genes that are induced by the natural flora are indeed implicated in defending against opportunistic bacterial infections, we assayed the proliferation of the mosquito midgut flora upon their silencing. We subjected 12 genes to this test of which *Cec1*, *Cec3*, *Def1*, *ClipA9*, *Gambicin*, *PGRP-LB*, and *FBN9* were induced by the presence of the natural bacterial flora, and the remaining *LRRD7*, *LRRD19*, *TEP1*, *Rel1* and *Rel2* genes represented anti-*Plasmodium* pattern recognition receptors or immune signaling pathway factors [35], [45]–[47]. Depletion of *Cec3*, *Gambicin*, *PGRP-LB*, *LRRD7*, *TEP1*, and *Rel2* resulted in the significant proliferation of the natural bacterial flora in the mosquitoes' midguts. Gene silencing of *Cec1*, *Def1*, *ClipA9*, *FBN9*, and *LRRD19* also resulted in some increase of bacterial loads in the midgut; however these effects were not statistically significant (Figure 6). The lack of significant bacterial proliferation in these knock-down mosquitoes could also be explained by the lower efficacy of gene silencing in the midgut tissue compared to the abdominal and thoracic compartment (Figure S3, Table S1). These results show that the mosquito's innate immune system is actively involved in controlling the bacterial load in the midgut lumen in a constitutive fashion, and that exposure to increased bacteria will result in increased production of some of these anti-*Plasmodium* factors. We believe that this is the mechanistic basis of how the mosquito's endogenous flora is important in priming an anti-*Plasmodium* defense.

### The anti-*Plasmodium* action of immune genes can be modulated by the presence of the mosquito's endogenous microbiota

**Figure 7:**
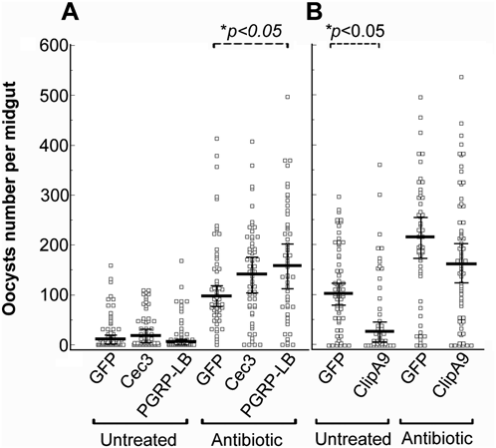
The depletion of PGRP-LB, Cec3, and ClipA9 through RNAi gene silencing resulted in the changes of *P. falciparum* oocyst intensity in the septic (untreated) and aseptic (antibiotic-treated) mosquitoes. Points indicate the absolute value of oocysts counts in individual mosquitoes, and horizontal black bars in each column represent the median value of oocysts from three replicates where the narrow black bars above or below the median values indicate the standard errors. *p*-values were calculated through a Kruskal-Wallis test. (A) *P. falciparum* oocyst intensity increased in aseptic mosquitoes (Antibiotic) when *Cec3* or *PGRP-LB* was silenced. *dsGFP* injected mosquitoes (GFP) were used as controls. (B) *P. falciparum* oocyst intensity decreased in septic mosquitoes (Untreated) when *ClipA9* was silenced.

The dual role of anti-*Plasmodium* factors in defending against both the parasite and bacteria, and the influence of bacteria on *Plasmodium* development, suggests the existence of complex interactions and relationships between the parasite, the microbiota and the mosquito's innate immune system. For example, the anti-*Plasmodium* activities of certain genes might be modulated by their parallel activities against bacteria. To assess such complexities and interactions, we studied the effect of various immune genes on *P. falciparum's* capacity to establish infection in the midgut tissue of both septic and aseptic mosquitoes through RNAi gene silencing approach (Figure 7).

RNAi-mediated depletion of the antimicrobial peptides Cec1, Def1, and Gambicin had no statistically significant effect on the levels of *P. falciparum* oocyst infection in either mosquito groups (data not shown), while gene silencing of *Cec3* and *PGRP-LB* resulted in an increased susceptibility to *P. falciparum* only in the aseptic mosquitoes (Figure 7A). This result may suggest that the depletion of these two immune genes in septic mosquitoes resulted in a proliferation of the microbial flora which in turn may have counteracted, or masked, the potential decrease of anti-*Plasmodium* immune responses.

Another striking example of how important the microbial flora is in regulating anti-*Plasmodium* activity of immune genes is represented by the serine protease ClipA9. When this factor was depleted in septic conditions, the mosquitoes became significantly less susceptible to *P. falciparum* infection (*p*<0.05). In contrast, when *ClipA9* was silenced in aseptic mosquitoes it had no significant effect on susceptibility to the parasite (Figure 7B). This observation suggests that the ClipA9-mediated anti-*Plasmodium* defense is exerted through the microbial flora and not directly against the parasite. ClipA9 is likely to be more specific for antibacterial defense and its depletion, under septic conditions, will hence result in the proliferation of bacteria which will exert strong anti-*Plasmodium* activity. Alternatively, it may mediate some direct *Plasmodium* protective activity which is abolished in the absence of bacteria. Interestingly the malaria parasite infection phenotype of ClipA9 gene silencing is opposite to that observed for the serine protease inhibitor SRPN6, suggesting that SRPN6 may function in the same cascade as an inhibitor of ClipA9 [30],[39].

In conclusion, similarly to humans, the mosquito intestine harbors a natural microbiota which is necessary for maintaining normal physiological functions including host metabolism and immune homeostasis. Accordingly, we have shown that the mosquito's natural bacterial flora show great variability between mosquitoes originating from the same colony and that it is an important regulator of mosquito permissiveness to *Plasmodium*. The mosquito's natural microbiota and artificially introduced non-natural bacteria negatively affected malaria parasite development through a mechanism that appears to implicate in the innate immune system, and not a direct killing of *Plasmodia* by the bacteria. The natural bacterial flora is essential in inducing a basal level immunity that in turn enhances the mosquito's ability in defending against the infection from the malaria parasites [35].

Interestingly, the effect of certain immune genes on *Plasmodium* infection is dependent on the presence of the microbial flora, suggesting that their mode of action is complex. This finding suggests that future studies on gene specific anti-*Plasmodium* action should also consider the complex interplay between the microbiota and the mosquito's immune defenses against the *Plasmodium* parasite. This relationship is further corroborated by observations from Dr. Barillas-Mury's group, where RNAi gene silencing of one immune gene facilitated the proliferation of microbial flora but reduced the *Plasmodium* infection.

The natural bacterial flora has also been shown to be involved in the suppression of other pathogenic organisms in other mosquito species. Tetracycline treatment of *Culex bitaeniorhynchus* rendered this mosquito more susceptible to the Japanese encephalitis virus [48] and the *Aedes aegypti* mosquito microbial flora has been shown to stimulate a basal-level immunity which suppresses dengue virus infection [25].

## Materials and Methods

### Ethics statement

All animals were handled in strict accordance with good animal practice as defined by the relevant national and/or local animal welfare bodies, and all animal work was approved by the appropriate committee.

### Mosquito rearing, antibiotic treatments, and RNA sample preparation

*A. gambiae* Keele strain mosquitoes were maintained on a 10% sugar solution in laboratory culture at 27°C and 70% humidity with a 12 hrs light/dark cycle according to standard rearing procedures [49]. A single cohort of adult female mosquitoes were collected immediately after eclosion, and either maintained under normal, non-sterile insectary conditions or placed into a sterile environment. Following, adult female mosquitoes were daily given fresh filtered sterilized 10% sucrose solution containing 15 µg gentamicin sulphate (Sigma) and 10 units/10 µg of penicillin-streptomycin (Invitrogen) per ml, respectively. Each cohort of mosquitoes was simultaneously membrane-fed freshly washed human erythrocytes resuspended to 40% haematocrit using human serum. As far as possible, every care was taken to maintain the sterility of the blood and membrane-feeding apparatus used to feed the mosquitoes, in order to prevent the antibiotic-treated mosquitoes acquiring bacterial infection during the process of membrane-feeding. The mosquitoes were starved for 8 hrs before feeding to encourage engorgement, and sugar solution was replaced once blood feeding had finished. At 24 hrs after blood feeding, 20 mosquitoes from each replicate of each cohort was collected and dissected on ice. RNA was extracted from dissected tissues at the assayed time points using the RNeasy kit (Qiagen). The quantification of RNA concentrations was performed using a Spectrophotometer (Eppendorf).

### Microarray hybridization and data mining

Probe sequence design and microarray construction were kept the same as described in [14]. Probe preparation and microarray hybridizations were performed essentially as previously described with some modifications [14]. Briefly, Cy3-labeled control cRNA probes and Cy5-labeled treatment cRNA probes were synthesized from 2–3 µg of RNA using the Agilent Technologies low-input linear amplification RNA labeling kit according to the manufacturer's instructions. Hybridizations were performed with the Agilent Technologies in situ hybridization kit according to the manufacturer's instructions with 2 µg of cRNA probes and 16 hrs after hybridization the microarray slides were washed and dried with compressed air. Microarrays were scanned with an Axon GenePix 4200AL scanner using a 10 µm pixel size (Axon Instruments, Union City, California, United States). Laser power was set to 60%, and the photomultiplier tube (PMT) voltage was adjusted to maximize effective dynamic range and minimize the saturation of pixels. Scanned images were analyzed by using GenePix software, and Cy5 and Cy3 signal and ratio values were obtained and subjected to statistical analysis with TIGR MIDAS and MeV software [50]. The minimum signal intensity was set to 100 fluorescent units, and the signal to background ratio cutoff was set to 2.0 for both Cy5 and Cy3 channels. Three or four biological replicates were performed for each experimental set. The background-subtracted median fluorescent values for good spots (no bad, missing, absent, or not-found flags) were normalized according to a LOWESS normalization method, and Cy5/Cy3 ratios from replicate assays were subjected to *t* tests at a significance level of *p*<0.05 using cutoff value for the significance of gene regulation of 0.7 and 0.8 in log2 scale, for septic mosquitoes and mosquitoes co-fed with experimental bacteria respectively, according to previously established methodology [51]. Microarray-assayed gene expression of 6 genes was further validated with quantitative RT-PCR and showed a high degree of correlation with the Pearson correlation coefficient (p = 0.84), the best-fit linear-regression analysis (R^2^ = 0.70), and the slope of the regression line (m = 1.247) demonstrated a high degree of correlation of the magnitude of regulation between the two assays (Figure S2).

### Primers design and qRT–PCR

Primers' sequences for validation of microarray hybridization data were as described in [14]. And new primers for RNAi gene silencing and verification were designed with Primer 3 Program on a web-based server (http://frodo.wi.mit.edu/). All the primer sequences were listed in Table S1. Real-time quantitative PCR (qRT–PCR) to check the efficiency of gene silencing were done essentially according to [14]. The quantification was performed using the QuantiTect SYBR Green PCR Kit (Qiagen) and ABI Detection System ABI Prism 7300. All PCR reactions were performed in triplicate. Specificity of the PCR reactions was assessed by analysis of melting curves for each data point. The ribosomal protein S7 gene was served as internal control for normalization of cDNA templates.

### RNAi gene silencing and *P. falciparum* infection assays

Sense and antisense RNAs were synthesized from PCR-amplified gene fragments using the T7 Megascript kit (Ambion). The sequences of the primers are listed in Table S1. *dsRNA* mediated gene silencing was done according to [14],[28]. About 80 4-d-old female mosquitoes were injected, in parallel, with GFP *dsRNA* as a control group or with target gene–specific *dsRNA* for the experimental group. Gene silencing in the whole mosquitoes was verified 3 to 4 d after *dsRNA* injection by qRT-PCR, done in triplicate, with the *A. gambiae* ribosomal *S7* gene as the internal control for normalization. Gene silencing efficiency were listed in Table S1 with standard errors shown (KD%±SE). RNAi gene silencing in the midguts was verified by RT-PCR, 10 midguts were used for each replicate and at least two replicates were included with only one replicate shown (Figure S3). At least 50 control (GFP *dsRNA*–injected) and 50 experimental (gene *dsRNA*–injected) mosquitoes were fed on the same *P. falciparum* NF54 gametocytes culture at 3–4 d after the *dsRNA* injection. 24 hrs post blood feeding (pbf), the unfed mosquitoes were removed and the fed-mosquitoes were dissected at 7–8 d after feeding and midguts were stained with 0.2% mercurochrome [43]. Oocyst numbers per midgut were determined using a light-contrast microscope (Olympus). The median number of oocysts per midgut was calculated for each tested gene and for GFP *dsRNA*–injected control mosquitoes. The results for equal numbers of midguts from all three independent biological replicates were pooled. The dot plots of the oocysts number in each midgut within each treatment were presented by MedCalc software with the median value of the oocysts indicated. The Kruskal-Wallis (KW) test and Mann-Whitney test were used to determine the significance of oocysts numbers (*p*<0.05).

### Co-feeding and pre-injection of bacteria and *P. falciparum* infection assays

About 80 4-day-old mosquitoes were first injected with PBS as control, or a mixture of live bacteria with approximately 30,000 *E. coli* and 60,000 *S. aureus*, or a mixture of heat-inactivated bacteria with the same number as the live ones. 24 hrs or 48 hrs after injection, mosquitoes were fed with *P. falciparum* NF54 gametocytes culture which were carried out according to our establish protocols [14]. For the co-feeding assay, the same sets of control PBS or bacteria were mixed in the blood meal to result in the same amount of either bacterium in the mosquito midguts. Unfed mosquitoes were removed, and the rest were kept in 26°C for 8 days before the oocysts counts. The infection phenotypes were determined as described above.

### Endogenous bacteria enumeration from mosquitoes' midguts

Isolation and colony forming units (CFU) enumeration of bacteria from midguts of untreated control, antibiotic-treated mosquitoes and gene-silenced mosquitoes were done essentially according to [43] with modifications. The midguts from surface sterilized mosquitoes were dissected with sterilized PBS 4 d after *dsRNA* injection, and CFU were determined by plating the homogenate of the midguts with series dilutions on LB agar plates and incubating the plates at 27°C for 2 days. Each assay was performed with one midgut and at least 10 independent replicates were included for each gene. The species of the isolated bacteria were determined by amplifying a region of the 16s rDNA as described by using primers 27f and 1492r [52]. PCR products were sequenced and blasted against Nucleotide collection (nr/nt) database to verify the species.

### Immuno-fluorescent microscopy of ookinetes from bloodmeal and oocysts from midgut epithelium

The early stages of *P. falciparum* development within untreated, antibiotic-treated and bacteria co-feeding mosquito midguts were compared by using the immuno-staining of ookinetes with anti-Pfs25 antibody (MRA-28, provided by MR4). Preparation of samples for immuno-fluorescence microscopy of malaria parasite within the bloodmeal was carried out based on [53] with substantial modifications. Sterile 0.5 ml “non-stick” low retention hydrophobic tubes (Alpha Laboratory Supplies) and sterile “non-stick” low retention hydrophobic pipette tips (Alpha Laboratory Supplies) were used to minimize malaria parasite loss during sample preparation due to their adhesion to plastic surfaces. The midguts including the entire bloodmeal contents were individually homogenized and diluted in 280 µl of PBS. 10 µl was then spotted, in duplicate, onto Teflon®-printed microwell glass slides (VWR International) previously coated with 3-aminopropyltriethoxysilane (APES) according to the supplier's instructions (Sigma). The sample slides were then air-dried, fixed in ice cold acetone for 2 mins and subjected to blocking in 10% goat serum for 1 hr, followed by the incubation with primary antibodies at 1∶400 dilutions for 2 hrs. After three PBS washes, sample slides were incubated with secondary antibodies (Molecular Probes, 1∶1000) for 2 hrs with Alexa Fluor 488-conjugated (green) goat anti-mouse antibody (1∶500 dilution). After another three PBS washes, sample slides were analyzed under a Nikon E800 upright microscope with epi-fluorescence. The total number of round forms, retort-forms and mature ookinetes in each spotted sample was counted. Average values for the densities of each malaria parasite stage present within each midgut examined were calculated from the three replicates. For checking the ookinetes and early oocysts in the midgut epithelium cells, at 24–30 hrs or 8 d after blood feeding, the midguts were dissected in 1% paraformaldehyde and washed with 3 times of PBS to remove the blood content and were subjected to the fixation in 4% paraformaldehyde (in PBS) for 1 hr and followed with 2 PBS washes. The midguts were then subjected to blocking and immune staining with primary antibody and secondary antibody as mentioned above. Midguts stained with pre-immune of anti-Pfs25 antibody were used as control. Midgut samples were mounted using the ProLong Antifade Kit (Molecular Probes) with DAPI staining of the cell nuclei and analyzed with same microscopy set as described above.

## Supporting Information

Figure S1 Survival rates of *A. gambiae* Keele mosquitoes after *P. falciparum* infection. At least 40 mosquitoes were in each replicate, and three replicates were included with standard errors shown. Non-treated: septic mosquitoes harbor natural microbiota; Antibiotic-treated: mosquitoes treated with antibiotics, referred as aseptic mosquitoes; Rechallenged: aseptic mosquitoes co-fed with bacteria and *P. falciparum* infected blood.
(0.02 MB PDF)

Figure S2 Validation of microarray-assayed gene expression with qRT-PCR. The values for the expression data obtained by microarray analysis (log2 ratio) for six genes were plotted against the corresponding expression values obtained with qRT-PCR (also log2 transformed) from two biological replicates of each experiment. Only the comparisons between the whole septic and aseptic mosquitoes which fed on sugar or uninfected blood were shown here.
(0.01 MB PDF)

Figure S3 Verification of gene silencing in the mosquito midgut tissue 4-d post *dsRNA* injection. *dsGFP*-injected mosquito midguts were used as controls, and 10 midguts were included in each replicate and at least two replicates were done with only one replicate shown here. Def1: *defensin 1*, Gam: *gambicin*; Cec: *cecropin*.
(0.09 MB PDF)

Table S1 Primers used for *dsRNA* synthesis, qRT-PCR validation of RNAi-mediated gene silencing and the efficiency of gene silencing.
(0.01 MB PDF)

Table S2 List of genes identified from microarray analysis exhibiting significant differential expression between untreated septic and antibiotic-treated aseptic adult female *A. gambiae* Keele mosquitoes fed with sugar (7-day-old whole mosquitoes minus head).
(0.06 MB XLS)

Table S3 List of genes identified from microarray analysis exhibiting significant differential expression between septic and aseptic adult female *A. gambiae* Keele mosquitoes fed on uninfected blood (7-day-old whole mosquitoes minus head).
(0.06 MB XLS)

Table S4 List of genes identified from microarray analysis exhibiting significant differential expression in the midguts of 7-day-old female *A. gambiae* Keele mosquitoes 12 hrs after feeding on uninfected blood supplemented with *E. coli* and *S. aureus*, PBS substitution of bacteria as control.
(0.17 MB XLS)

Table S5 Table S5. List of genes identified from microarray analysis exhibiting significant differential expression in the carcass of 7-day-old female *A. gambiae* Keele mosquitoes 12 hrs post feeding on uninfected blood supplemented with *E. coli* and *S. aureus*, PBS substitution of bacteria as control.
(0.15 MB XLS)
