# Supplementary figures and images for: Implication of the Mosquito Midgut Microbiota in the Defense against Malaria Parasites
Source: PLoS Pathog. 2009 May 8;5(5):e1000423. doi: 10.1371/journal.ppat.1000423 (PMC2673032; doi:10.1371/journal.ppat.1000423)

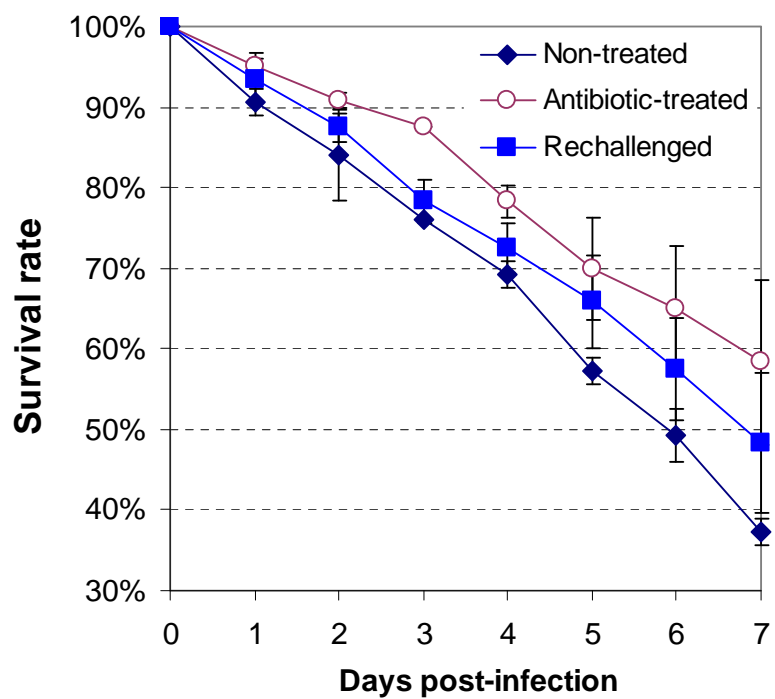

Supplement: Figure S1 — Survival rates of A. gambiae Keele mosquitoes after P. falciparum infection. At least 40 mosquitoes were in each replicate, and three replicates were included with standard errors shown. Non-treated: septic mosquitoes harbor natural microbiota; Antibiotic-treated: mosquitoes treated with antibiotics, referred as aseptic mosquitoes; Rechallenged: aseptic mosquitoes co-fed with bacteria and P. falciparum infected blood. (0.02 MB PDF) [file ppat.1000423.s001.pdf]

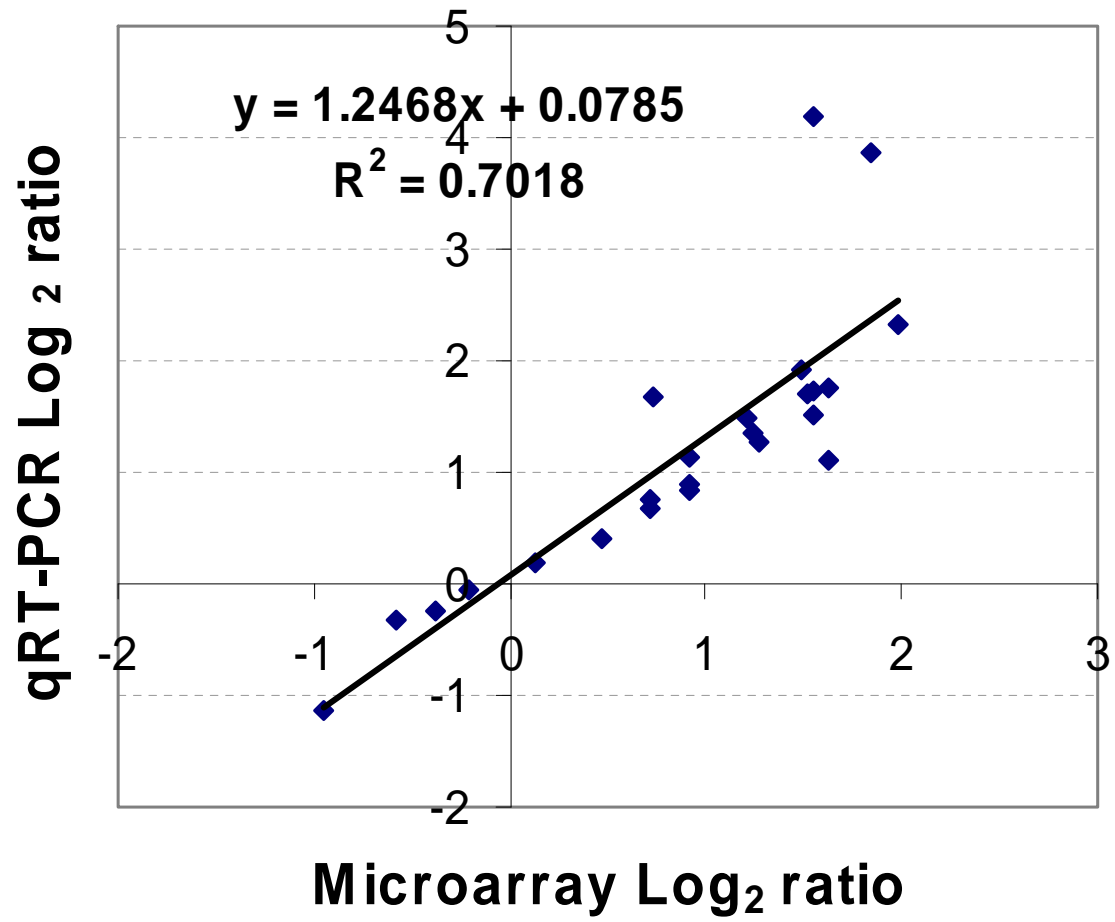

Supplement: Figure S2 — Validation of microarray-assayed gene expression with qRT-PCR. The values for the expression data obtained by microarray analysis (log2 ratio) for six genes were plotted against the corresponding expression values obtained with qRT-PCR (also log2 transformed) from two biological replicates of each experiment. Only the comparisons between the whole septic and aseptic mosquitoes which fed on sugar or uninfected blood were shown here. (0.01 MB PDF) [file ppat.1000423.s002.pdf]

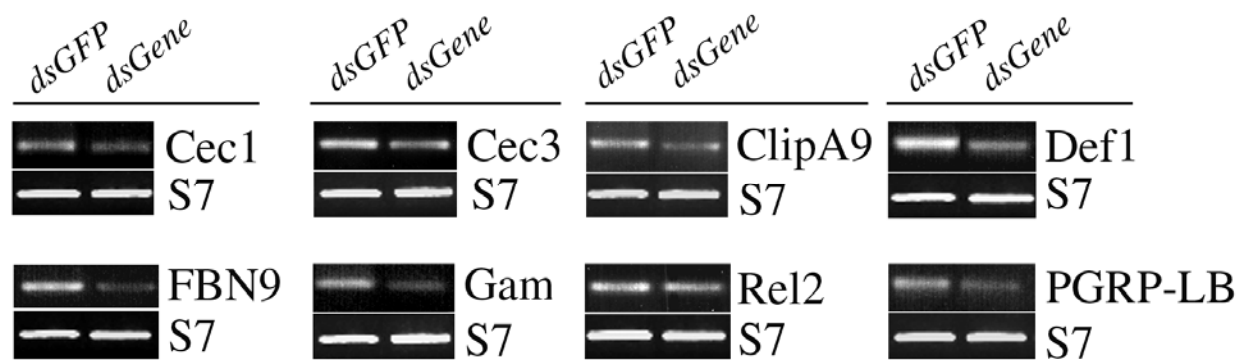

Supplement: Figure S3 — Verification of gene silencing in the mosquito midgut tissue 4-d post dsRNA injection. dsGFP-injected mosquito midguts were used as controls, and 10 midguts were included in each replicate and at least two replicates were done with only one replicate shown here. Def1: defensin 1, Gam: gambicin; Cec: cecropin. (0.09 MB PDF) [file ppat.1000423.s003.pdf]
