# Supplementary material for: Implication of the Mosquito Midgut Microbiota in the Defense against Malaria Parasites
Source: PLoS Pathog. 2009 May 8;5(5):e1000423. doi: 10.1371/journal.ppat.1000423 (PMC2673032; doi:10.1371/journal.ppat.1000423)
Supplement: Table S1 — Primers used for dsRNA synthesis, qRT-PCR validation of RNAi-mediated gene silencing and the efficiency of gene silencing. (0.01 MB PDF) [file ppat.1000423.s004.pdf]

Table S1. Primers used for *dsRNA* synthesis, qRT-PCR validation of RNAi-mediated gene silencing and the efficiency of gene silencing.

Gene Name	Primer Name	ENSAN GT#	KD% (± SE)	Primer sequence
Cecropin1	Cec1RNAiF	#11957	72 (±13)	TAATACGACTCACTATAGGG ATCAACCCAGAGACCAACCA
	Cec1RNAiR			TAATACGACTCACTATAGGGGCTGATTCATCTCTCTCCCTCT
	Cec1veriR			GAAGTGAAATTTATTTGCCGAAC
Cecropin3	Cec3RNAiF	#11995	67 (±13)	TAATACGACTCACTATAGGGCATCTGTGTCCAAAACCAA
	Cec3RNAiR			TAATACGACTCACTATAGGATTGTCGATGCAGCTGTTTG
	Cec3veriF			GAGATCTCTTCCCGTGTGGA
ClipA9	ClipA9RNAiF	#12706	88 (±8)	TAATACGACTCACTATAGGGATCGCTGTCAGCTGTTGTATCGA
	ClipA9RNAiR			TAATACGACTCACTATAGGCCGCAACGTTACAAATGTTC
	ClipA9veriF			TGCTGAAACGCAACGTAATC
Defensin1	Def1RNAiF	#15621	68 (±14)	TAATACGACTCACTATAGGGCTGTGCCTTCCTAGAGCATC
	Def1RNAiR			TAATACGACTCACTATAGGGCACACCCTCTTCCCAGGAT
	Def1veriR			AATGCATGGACACGAATGAA
Gambicin	GambRNAiF	#13255	80 (±12)	TAATACGACTCACTATAGGGCTATCTCAACCGGAAGG
	GambRNAiR			TAATACGACTCACTATAGCCAACGTCTGGCACTGATT
	GambveriF			CGGCAGCCGTTGCGGATGCAATG
PeritrophinA	PeriARNAiF	#13237	58 (±9)	TAATACGACTCACTATAGGGTTCTGCCAGTGTGGTGCTAC
	PeriARNAiR			TAATACGACTCACTATAGGGCGTACGGATCGCAAATGTAA
	PeriAveriR			CAACTCTGGGAAGTCGCAAT
PGRP-LB	PGRPLBRNAiF	#13948	77 (±7)	TAATACGACTCACTATAGGTACGTGACGCGAGACTTTTG
	PGRPLBRNAiR			TAATACGACTCACTATAGGCCCCAGCAAGGTGTAGTTCT
	PGRPLBveriR			GGCCAGGTTTTGATCTCCTC
LRRD7	LRRD7-F	#21822	80 (±11)	TAATACGACTCACTATAGTCGGTGAGCAACAGTTTGAC
	LRRD7-R			TAATACGACTCACTATAGCTTCATTCCCGCTAATGCTC
	LRRD7-veriF			CGCCACGATCGAAAGCACCGCGT
FBN9	FBN9-F	#11248	66 (±18)	TAATACGACTCACTATAGCCAAGATGTCGGGCAAGTAT
	FBN9-R			TAATACGACTCACTATAGTTGTGGTACGTCAGCGAGTC
	FBN9-veri			CGCTCCCTGTTCGAGCTGCA
Tep1	Tep1-F	#16857	78 (±9)	TAATACGACTCACTATAGGTTTGTGGGCCTTAAAGCGCTG
	Tep1-R			TAATACGACTCACTATAGGACCACGTAACCGCTCGGTAAG
	Tep1-veriF			GGTGAATCAACGGTACGTTA

KD% (± SE): RNAi gene silencing efficiency with standard errors; ENSANGT#: last 5 digits from Transcript accession number from Ensembl.
